# Complete Genome Sequences of the First Reported California H16 Influenza A Viruses

**DOI:** 10.1128/genomeA.00329-14

**Published:** 2014-05-01

**Authors:** LeAnn L. Lindsay, Magdalena Plancarte, Maris Brenn-White, Walter M. Boyce

**Affiliations:** Department of Pathology, Microbiology and Immunology, University of California, Davis, California, USA

## Abstract

Two reassortant H16 influenza A viruses were isolated from gulls in California. Seven of the eight segments were most closely related to H16 and H13 isolates from eastern North America and Iceland. Of note is a C-terminal truncation of the nonstructural 1 (NS1) protein in one of the isolates that is usually found in swine H1N1 virus.

## GENOME ANNOUNCEMENT

Wild birds are a natural reservoir for influenza A viruses, and in several instances, avian influenza has been the source of influenza epidemics in other species (1). Avian influenza (AI) viruses are classified into 16 hemagglutinin (HA) and nine neuraminidase (NA) subtypes, with H13 and H16 largely restricted to gulls/shorebirds (*Charadriiformes*) (2, 3). Reports of H13 and H16 virus isolates are relatively uncommon compared to subtypes found in waterfowl, most likely due to sampling efforts being focused on ducks and geese. Of the ~100 H13 and H16 viruses isolated from North America, most have come from the Atlantic coast and Alaska. To date, no isolates have been reported for the U.S. Pacific coast south of Alaska.

In March of 2012, fresh fecal droppings from approximately 1,750 gulls were sampled throughout the California coastal region, and one avian influenza virus isolate was obtained from a California gull on Catalina Island near Los Angeles. In July of 2013, 300 California gull chicks were sampled at Mono Lake in the California Sierra Nevada mountain range, and one isolate was obtained from a cloacal swab. Through next-generation deep sequencing, all eight segments of the two isolates were sequenced, and the subtype was determined to be H16N3. The isolates were named A/env/California/1242V/2012 (H16N3) and A/California gull/California/1196P/2013 (H16N3).

Phylogenetic analysis of the two isolates showed that seven of the eight genome segments were shared. The HA, polymerase acidic (PA), polymerase basic 1 (PB1), and PB2 genome segments were most similar to those of A/black headed gull/Iceland/713/2010 (H16N3) (98 to 99% nucleotide identity), while the nucleoprotein (NP) segments were most similar to those of A/ruddy turnstone/New Jersey/AI09-294/2009 (H13N6) (98%). The matrix (M) segments were most similar to those of A/ring-billed gull/Quebec/02622-1/2009 (H3,13N6) (99%), and the NA segments were most closely related to those of A/glaucous-winged gull/Southeastern Alaska/10JR01572R0/2010 (H16N3) (99%). The nonstructural (NS) segments for the two isolates are of different origins (94% nucleotide identity), with the A/env/California/1242V/2012 (H16N3) NS segment being most similar to the A/black headed gull/Iceland/713/2010 (H16N3) NS segment (99%) and the A/California gull/California/1196P/2013 (H16N3) NS segment closest to the A/herring gull/New Jersey/AI09-21105/2009 (H13N6) segment (99%). Overall, the isolates are unique reassortants of varied North American H16 and H13 AI viruses.

Interestingly, the A/env/California/1242V/2012 (H16N3) NS1 amino acid sequence showed a truncation of 11 amino acids at the C-terminal end not seen in any other H16 viruses in the GenBank database. Various C-terminal truncations of the influenza virus NS1 protein have been reported, including in pandemic H1N1, but overall, it is very uncommon (4). The NS1 protein is involved in regulating gene expression and plays a role in inhibiting the host antiviral response, namely through inhibiting interferon synthesis. The truncation of NS1 does not appear to have an effect on the replication of the virus; however, the inhibition of host gene expression is impaired, and increased virulence in mice has been demonstrated (5). Since the A/env/California/1242V/2012 (H16N3) NS1 amino acid sequence is nearly identical to the 2010 Icelandic sequence (1 amino acid difference), apart from the truncation, the A/env/California/1242V/2012 (H16N3) C-terminal truncation appears to be a recent mutation resulting in a premature stop codon. The possible functional impacts of this mutation merit continued investigation.

### Nucleotide sequence accession numbers.

The genome sequences are available in GenBank under accession no. CY176997 to CY177004 and CY177441 to CY177448.
